# Biogeographical Patterns and Assembly of Bacterial Communities in Saline Soils of Northeast China

**DOI:** 10.3390/microorganisms10091787

**Published:** 2022-09-05

**Authors:** Xiaolong Liang, Xiaoyu Wang, Ning Zhang, Bingxue Li

**Affiliations:** 1 Key Laboratory of Pollution Ecology and Environmental Engineering, Institute of Applied Ecology, Chinese Academy of Sciences, Shenyang 110016, China; 2 College of Land and Environment, Shenyang Agricultural University, Shenyang 110866, China; 3 College of Bioscience and Biotechnology, Shenyang Agricultural University, Shenyang 110866, China

**Keywords:** bacterial community, soil pH, latitude gradient, salinity–sodicity, soil degradation, biogeographical pattern

## Abstract

Increasing salinity undermines soil fertility and imposes great threats to soil ecosystem productivity and ecological sustainability. Microbes with the ability to adapt to environmental adversity have gained increasing attention for maintenance and restoration of the salt-affected soil ecosystem structure and functioning; however, the characterization of microbial communities in saline–sodic soils remains limited. This study characterized the bacterial community composition and diversity in saline–sodic soils along a latitude gradient across Northeast China, aiming to reveal the mechanism of physicochemical and geographic characteristics shaping the soil bacterial communities. Our results showed that the bacterial community composition and diversity were significantly impacted by soil pH, electrical conductivity, Na^+^, K^+^, Cl^−^, and CO_3_^2−^. Significant differences in bacterial diversity were revealed along the latitude gradient, and the soil factors accounted for 58.58% of the total variations in bacterial community composition. Proteobacteria, Actinobacteria, Gemmatimonadetes, Chloroflexi, and Bacteroidetes were dominant across all samples. Actinobacteria and Gemmatimonadetes were significantly enriched in high soil sodicity and salinity, while Acidobacteria and Proteobacteria were suppressed by high pH and salt stress in the saline–sodic soils. Increase in soil pH and salinity significantly decreased bacterial species richness and diversity. Community composition analysis indicated that bacterial taxonomic groups (e.g., *Bacillus*, *Egicoccus*, *Truepera*, *Halomonas*, and *Nitrolancea*) that may adapt well to high salinity were greatly enriched in the examined soils. The findings collectively evidenced that bacterial community composition and diversity in a broad biographic scale were determined by niche-based environmental characteristics and biotic interactions. The profiling of the soil bacterial communities along the latitude gradient will also provide a basis for a better understanding of the salt-affected soil ecosystem functioning and restoration of these soil ecosystems.

## 1. Introduction

Soil salinization refers to the process of soil excessively accumulating soluble salts from various sources, which severely undermines soil fertility and imposes great threats to soil ecosystem productivity and ecological sustainability. Globally, a growing body of soil ecosystems are suffering from salinization because of unsustainable agricultural practices, industrial pollution, and natural processes [1,2]. With the rapidly increasing population and rising food demand, tremendous efforts have been made in prevention of soil salinization and restoration of the salt-affected soil ecosystem structure and function [3,4,5]. The impacts of salinization-induced soil degradation on plant physiology and productivity have been intensively investigated, and the study of microbiome in the saline soils has also gained increasing interest.

The microbial community, highly abundant and diverse in soil systems, has critical roles in maintaining the soil ecosystem structure and functioning. Many microbes can adapt to environmental adversity and directly engage in major ecosystem processes, such as nutrient cycling, pollutant transformation, and habitat modification under the environmental stresses [6,7,8]. Soil microbes may form complicated interactions (e.g., mutualism, competition, predation, commensalism, and parasitism) with the co-occurring other organisms in soil. The elements, including C, N, P, and K, contained in microbial cells are an important part of the soil nutrient pools [9], and microbial metabolic activities are important in regulating the biogeochemical cycling, such as carbon turnover in soil ecosystems [10,11,12]. Investigation of the geographic distribution and function of microbial communities in saline soils can provide insights into sustainable land management and restoration of salt-affected soil ecosystem structure and function.

Salinity is a critical factor for microbial physiology and metabolism, and changes in salinity can significantly affect microbial activities and influence the microbial community composition [13,14]. The extreme environmental conditions may suppress many microbial species but can provide a suitable environment for the salt-tolerant microorganisms, which may dominate in the saline soil environment [15,16]. With the functions of microbial communities in salt-affected soils being increasingly recognized, more efforts have been made in deciphering the role of salinity in shaping the structure and function of the soil microbiome. For example, an investigation of bacterial community structure and function natural salinity gradient showed that different groups of nitrifying and denitrifying bacteria had contrasting preference or sensitivity to salinity, and salinity was a key factor in shaping the bacterial community structure and processes, therefore influencing the total ecosystem services of the coastal wetlands [17]. A similar study in coastal estuarine wetlands suggested that salinity not only impacted the bacterial community composition, but also had a negative correlation with most functional genes, including genes associated with carbon degradation and nitrogen cycling [18]. Increased salinity greatly affects soil pH and other soil properties, which may also serve as key factors in shaping microbial cellular function and community structure [13,14,19]. Climatic conditions, such as air temperature and precipitation, in a region may be related to soil salinization and have major impacts on bacterial community diversity. The patterns and assembly of microbial communities on broad-scale geographic gradients remain limited. It also needs to be verified whether specific bacterial taxonomic groups may better adapt to high salinity and gain higher abundances within soils across different geographic locations. Therefore, more efforts are needed to reveal the mechanism of soil physiochemical and geographic characteristics driving microbial community structure.

The Northeast China Plain, with more than 3.7 million ha salt-affected soils, is one of the major grain-producing regions most severely impacted by soil salinization [20]. The widespread region includes various sorts and degradation degrees of salt-affected soils, which could serve as an ideal natural environment for studying soil microbial community under salinization. The aim of this study is to address the forementioned knowledge gap by a latitude-gradient field study of the saline soil ecosystems across the Northeast China Plain, including Songliao River Basin, the alluvial plain along Liao River, and coastal wetlands in Liaohe Delta. The bacterial community structure and soil physiochemical properties in all samples were examined, and the correlation of bacterial community composition with soil properties and geographic location was also investigated to reveal the environmental factors driving the bacterial communities in saline–sodic soils. The detailed profiles of the saline–sodic soil bacterial communities were unveiled to imply the major functional bacterial taxonomic groups within the extreme habitats. The hypotheses that we intended to address included: (i) salinity–sodicity would be the major factor in shaping bacterial community diversity and composition; (ii) geographic patterns of soil bacterial community may form along the latitude gradient across the Northeast China Plain; and (iii) the indicator bacterial taxa in the salt-affected soils would be notably different from other types of soil ecosystems.

## 2. Materials and Methods

### 2.1. Study Area and Field Sampling

**Figure 1:**
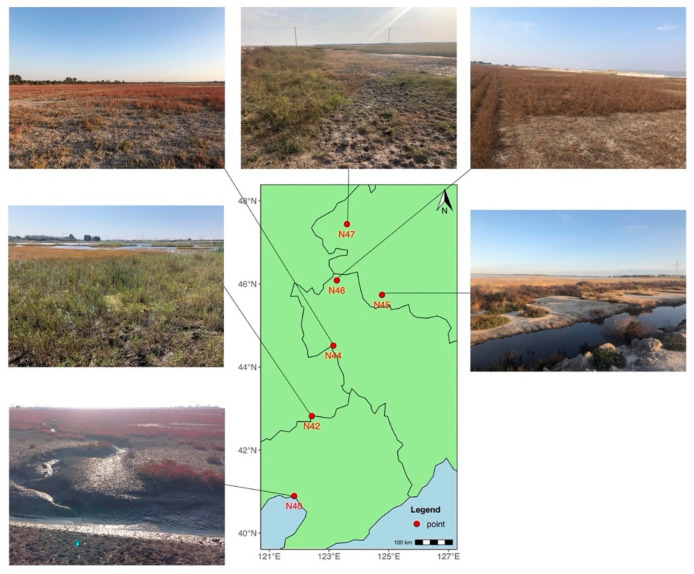
The map and profiles of the study area. Soil samples were collected from six sites (named N40, N42, N44, N45, N46, and N47, each representing the co-ordinates of the sampling site) across three provinces in Northeast China. The established study area consists of a 690-km south–north latitudinal gradient (40°53′ N to 47°26′ N).

The established study area consists of a 690-km south–north latitudinal gradient (40°53′ N to 47°26′ N) across three provinces in Northeast China (Figure 1). The sampling sites were in Zhangwu County (Liaoning Province), Panjin (Liaoning), Changling County (Jilin), Zhenlai County (Jinlin), Zhaoyuan County (Heilongjiang), and Qiqihaer (Heilongjiang), with the altitude ranging from 20 to 251 m (Table S1). The climate in the study area belongs to temperate monsoon climate, with an average annual air temperature of 5 °C, and the annual precipitation is around 520 mm and declines from southeast to northwest. Soil samples were collected from 18 locations in September 2019, and triplicate samples were collected from 3 different sites at each location. The surface soils at a depth of 0–20 cm were sampled. Each soil sample was collected from 20 sampling points with the sampling core, and the soils at each site were thoroughly mixed into one composite sample before being packaged into a polyvinyl chloride bag and kept in the cooler with ice. The samples were immediately shipped back to the laboratory, where the soil samples were archived at −80 °C until DNA extraction and further analysis of soil physiochemical properties.

### 2.2. Soil Physicochemical Properties Analysis

The soil samples were air dried and sieved through test sieves (mesh size: 2 mm; material: stainless steel; diameter: 203.2 mm). For measurement of soil pH, 10 g of dry soils were mixed with 10 mL 0.01 M CaCl_2_ solution, and the mixture was shaken for 1 h before pH measurement with Oakton Ion 700 benchtop pH meter. Soil electrical conductivity (EC) was determined in a soil–water mixture with a dry soil-to-distilled water ratio of 1:5 (*w*/*v*), and the temperature and soil electrical conductivity were recorded by conductivity meter [21]. Soil soluble salt content, including Na^+^, K^+^, Ca^2+^, Mg^2+^, CO_3_^2−^, HCO_3_^−^, Cl^−^, and SO_4_^2−^, were measured by methods described previously [22]. Na^+^ and K^+^ were measured in the soil extract from 1:5 soil–water mixture using flame photometer, and Ca^2+^ and Mg^2+^ were measured with atomic absorption spectrophotometer.

### 2.3. Soil DNA Extraction and 16S rRNA Gene Sequencing

The soil whole DNA was extracted from each soil sample by using DNeasy PowerSoil Pro Kit (Qiagen, Hilden, Germany), and the concentration of the extracted DNA solutions was measured with Quant-iT PicoGreen Assay Kit (Invitrogen, Carlsbad, CA, USA). The V3-V4 region of bacterial 16S rRNA gene was amplified by PCR using the barcoded primers of 338F (5’-ACTCCTACGGGAGGCAGCAG-3’) and 806R (5’-GGACTACHVGGGTWTCTAAT-3’). In PCR reactions, each 25 μL mixture contained a final concentration of 1 × Q5 reaction buffer, 2 mM dNTP, 0.4 μM forward primer, 0.4 μM reverse primer, 0.8 ng/μL template DNA, and 0.02 units/μL Q5 High-Fidelity DNA Polymerase (New England BioLabs Inc., Ipswich, MA, USA). The thermal program used for amplification was as follows: 98 °C for 2 min; followed by 30 cycles of 98 °C for 15 s, 55 °C for 30 s, 72 °C for 30 s; final extension at 72 °C for 5 min. The PCR products were run on 1.2% agarose gel electrophoresis, and the target sequences were purified with Agarose Gel DNA Purification kit (TaKaRa Biotechnology, Dalian, China). The purified PCR products were quantified, and an equimolar amount of the PCR products from each sample was pooled and sequenced via 300PE (paired-end) on Illumina MiSeq platform at Shanghai Personal Biotechnology Co., Ltd., Shanghai, China.

### 2.4. Bacterial Community Composition Analysis

The raw sequence files were demultiplexed and merged into contigs using FLASH software [23]. The merged contigs were sorted into each sample based on the unique 8-nucleotide barcode, and the sequences in each sample were then analyzed with QIIME2 DADA2 pipeline (version 1.16) [24]. The quality profiles of the reads were checked, with low-quality reads being filtered and trimmed to get rid of sequencing errors. Identification of chimeras was performed to remove the chimeric sequences. The quality-filtered sequences were clustered into OTUs at a similarity cutoff value of 97%. Taxonomic assignment to each of the 16S rRNA gene sequences was performed with naive Bayesian classifier method. The raw bacterial 16S rRNA gene sequences were uploaded to Sequence Read Archive (SRA) at NCBI and can be accessed via accession number of PRJNA752985.

### 2.5. Statistical Analysis

Rarefaction curves were evaluated in the “vegan” R package (version 2.5-7; developed at GitHub; website: https://github.com/vegandevs/vegan/) with maximum read depth to test if sufficient sequencing depth was achieved for representative coverage of the bacterial community diversity in each sample. The Chao1 index and species number were calculated to estimate bacterial species richness, and Shannon index was calculated to assess bacterial community diversity [25]. Distance-based redundancy analysis (db-RDA) was used to evaluate the impact of explanatory variables (i.e., soil physiochemical properties) on the bacterial community composition. Before RDA, the function of “ordiR2step” in vegan was used to select significant factors, which performs forward model choice based on adjusted R^2^_adj_ and *p*-value of permutation tests for explanatory variables in shaping the bacterial community composition. Variance partitioning analysis (VPA) was applied to evaluate the importance of environmental and PCNM factors using “varpart” function in vegan. Bray–Curtis dissimilarity was used for calculating the compositional dissimilarities of the bacterial communities from different saline–sodic soil samples. Linear analyses were performed to test the correlation of Bray–Curtis dissimilarity with differences in soil pH, Na^+^ content, geographic distance, and latitude. Mantel test was performed in the “ade4” R package (version 3.1.2) [26] for examining the effects of soil physicochemical properties on bacterial community composition. Spearman’s correlation analysis was conducted to reveal the correlations between different soil characteristics. A heatmap showing the distribution patterns of the 20 most abundant genus-level OTU groups was constructed with the dendrogram showing the cluster dissimilarity between samples based on maximum distance. Linear discriminant analysis effect size (LEfSE), utilizing Wilcoxon signed-rank test, Kruskal–Wallis H test, and linear discriminant analysis, was implemented in R for identification of the biomarker taxonomic groups within the bacterial community of each soil sample [27].

## 3. Results

### 3.1. Soil Physiochemical Properties

The soil physiochemical characteristics varied widely across the samples and are summarized in Table S2. Soil pH ranged from 8.13 to 10.45, which was lowest at N40 and highest at N45. The EC was highly varied across the soil samples, and soil EC was highest in the N44 soil samples (averaging 352 mS/cm) and lowest in the N42 soil samples (averaging 8.9 mS/cm). The N47 soils had the highest concentration of total Ca^2+^, while the N40 soils contained the most Mg^2+^ and K^+^. Na^+^ was the primary cation across all soil samples, with concentration ranging from 0.2‰ to 5.1‰ (*w*/*w*), while HCO_3_^-^ (ranging from 0.9‰ to 7.5‰) and SO_4_^2−^ (ranging from 2.3‰ to 7.3‰) were the most represented anions. The soil samples at N44 and N45 had significantly higher concentration of HCO_3_^−^ than other soil samples (*p* < 0.05; *t*-test), and the pH in N44 and N45 soils was significantly higher than other soils. N44 soil samples had the highest EC (352 mS/cm), followed by N45 (346 mS/cm), N40 (174 mS/cm), N47 (84 mS/cm), N46 (17 mS/cm), and N42 (9 mS/cm) soils. The high soil pH in the saline–sodic soils was primarily caused by a high concentration of HCO_3_^-^. Spearman’s correlation analysis showed that both soil pH and EC were significantly correlated with the content of HCO_3_^-^ and Na^+^ in soil (Spearman’s correlation analysis, *p* < 0.05; Figure S1). The concentration of Na^+^ was positively correlated with the content of CO_3_^2−^ (*p* < 0.01).

### 3.2. Soil Bacterial Community Diversity and Association with Soil Characteristics

**Figure 2:**
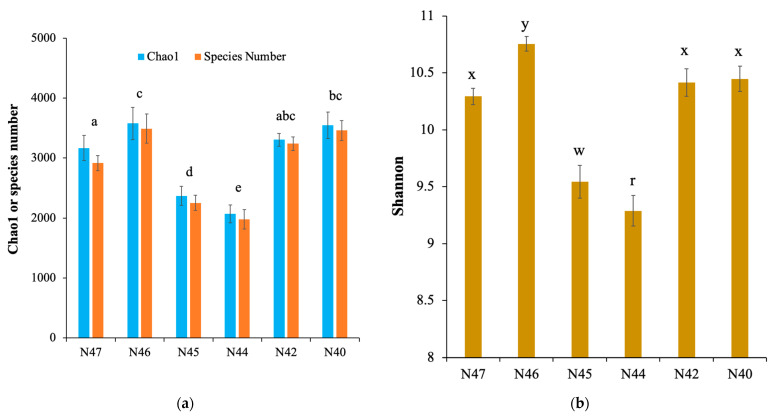
Bacterial community diversity in the saline–alkaline soil samples. (**a**) Bacterial species richness, represented by species number, in each sample. (**b**) Bacterial diversity, indicated with Shannon index, in each soil sample. Letters indicate significant differences according to Student’s *t*-test.

**Figure 3:**
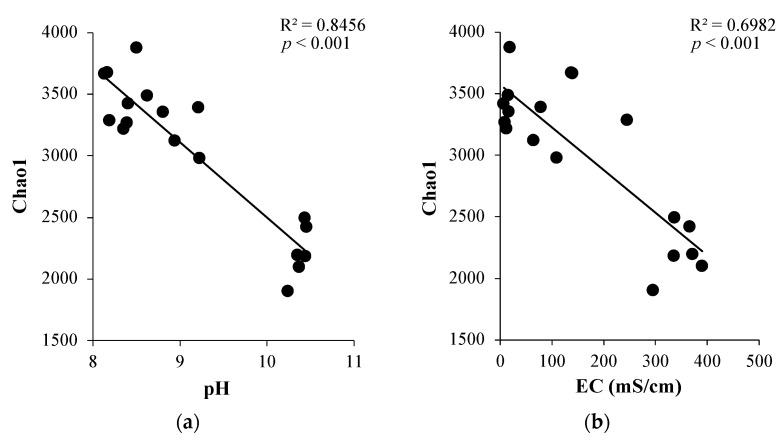

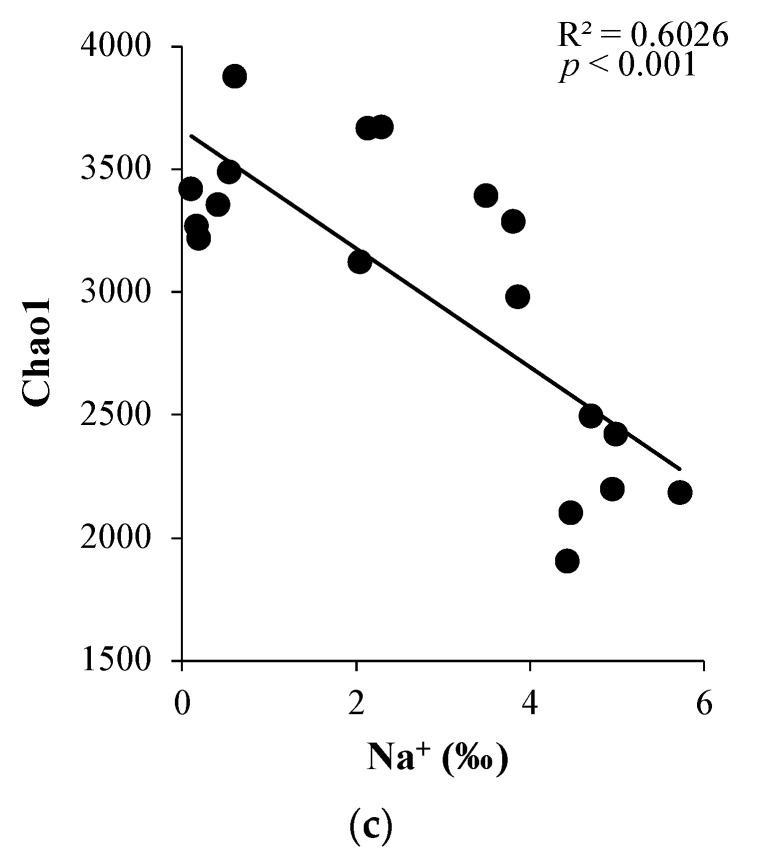
Linear regression between bacterial species richness and soil physiochemical properties. (**a**) Relationship between bacterial species richness (Chao1) with soil pH. (**b**) Relationship between bacterial species richness with soil EC. (**c**) Relationship between bacterial species richness with Na^+^ concentration in soil.

Rarefaction curves with maximum read depth showed that sufficient sequencing depth was achieved for representative coverage of the bacterial community diversity in each sample (Figure S2). The curves tend to be flat with the sequencing depth increasing, which suggests that the sequencing data volumes achieved in this study generated enough OTUs for further analysis. The soil samples at N44 had the lowest bacterial diversity and species richness, and the bacterial community diversity ranking was N46 > N40 > N42 > N47 > N45 > N44 (Figure 2). With the exception of the bacterial diversity in N40, N42, and N47 showing no statistically significant difference, the bacterial community diversity in all other samples was significantly different from each other. Both bacterial community diversity (*r* = −0.92; *p* < 0.001) and species richness (*r* = −0.9; *p* < 0.001; Figure 3) were negatively correlated with soil pH. Similarly, soil EC had negative correlations with the bacterial diversity (*r* = −0.84; *p* < 0.001) and species richness (*r* = −0.89; *p* < 0.001). The bacterial diversity and species richness were negatively correlated with the concentration of Na^+^, CO_3_^2+^, HCO_3_^−^, and Cl^−^ across all the soil samples (Table S3).

**Figure 4:**
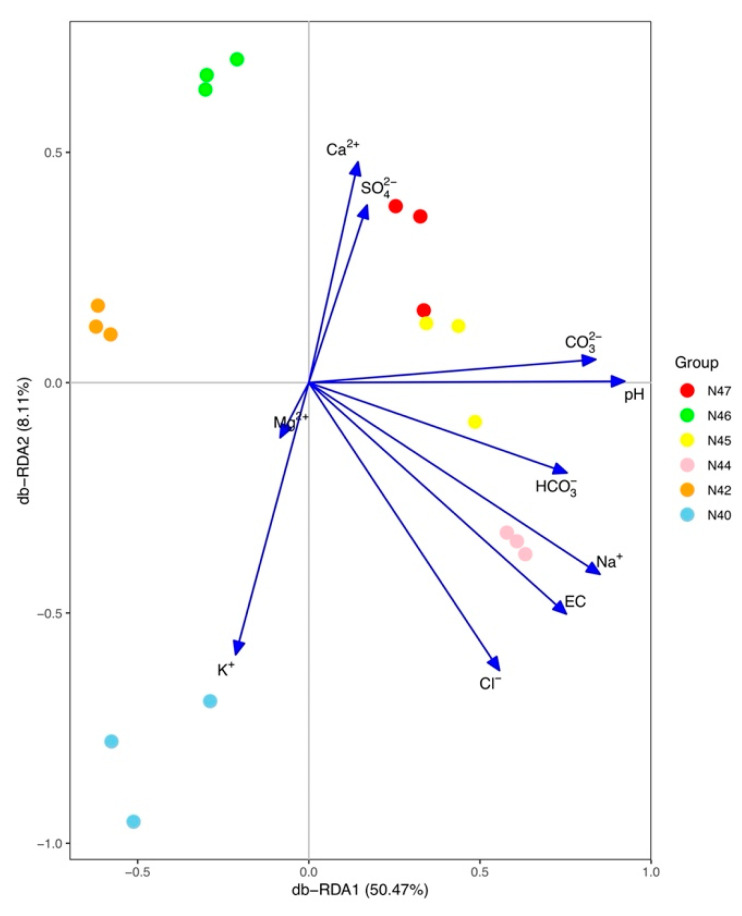
The bacterial beta diversity based on distance-based redundancy analysis (db-RDA) of driving factors of bacterial community composition. The environmental factors are plotted as vectors, and the length of the vector represents the magnitude of the impacts. The angles between vectors reflect their (linear) correlation.

**Figure 5:**
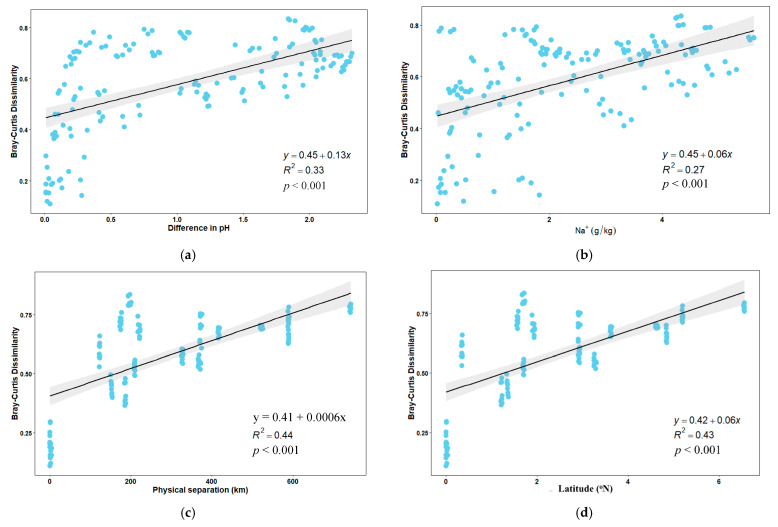
Correlation of Bray–Curtis dissimilarity of bacterial community composition with soil pH, Na^+^ content, geographic distance, and latitude. (**a**) Correlation between Bray–Curtis dissimilarity and soil pH. (**b**) Correlation between Bray–Curtis dissimilarity and Na^+^ concentration. (**c**) Correlation between Bray–Curtis dissimilarity and physical separation. (**d**) Correlation between Bray–Curtis dissimilarity and latitude of soil sampling.

Redundancy analysis (RDA) showing the dissimilarities of bacterial community composition was performed, and the major driving factors of bacterial community composition were also included as vectors in the RDA analysis. The results showed that all the samples collected from the same site clustered together and separated from all other samples (Figure 4). RDA analysis revealed that soil pH, EC, Na^+^, K^+^, Cl^−^, and CO_3_^2−^ were significant environmental factors shaping the bacterial community composition in the saline–sodic soils. RDA1 and RDA2 accounted for 58.99% and 9.94% of the total variations in bacterial community composition, respectively. The results of VPA suggested that PCMN factors explained 6.4% of the total variance of bacterial community composition, while environmental factors explained 47.5% of the toral variance. The interactions between environmental and PCMN factors contributed to 38.6% of the compositional dissimilarities of the bacterial communities. The bacterial community composition was more correlated with environmental factors than PCMN factors. The Bray–Curtis dissimilarity was significantly correlated with soil pH (*R^2^* = 0.33, *p* < 0.001), Na^+^ content (*R^2^* = 0.27, *p* < 0.001), geographic distance (*R^2^* = 0.44, *p* < 0.001), and latitude (*R^2^* = 0.43, *p* < 0.001; Figure 5).

### 3.3. Soil Bacterial Community Composition

The composition of the bacterial communities varied across the soil samples of different locations. Proteobacteria, Actinobacteria, Gemmatimonadetes, Chloroflexi, Bacteroidetes, Acidobacteria, and Firmicutes were the most abundant bacterial phyla in the soil samples (Figure S3). Proteobacteria was the most abundant bacterial phylum in the saline soils, and the relative abundance of Proteobacteria was highest in N40 soil samples (52.4%), followed by N42 (49.5%), N46 (36.7%), N47 (23.3%), N45 (22.1%), and N44 (19.1%) soil samples. Actinobacteria was the most abundant bacterial phylum in N44 soil samples, and the N44 soils had higher relative abundance of Actinobacteria (26.4%) than other soil samples. The relative abundance of Actinobacteria ranged from 12.6% to 26.4% across different samples. Gemmatimonadetes had a distribution pattern similar to Actinobacteria, and the relative abundance of Gemmatimonadetes was highest in N44 soil samples (24.5%), followed by N45 (20.2%), N47 (17.3%), N46 (9.1%), N40 (7%), and N42 (3%) samples. Chloroflexi and Bacteroidetes were relatively evenly distributed within different soil samples, as their relative abundances had a narrow range (Chloroflexi ranging from 7% to 15.4%; Bacteroidetes ranging from 7.5% to 12.4%). N46 soil samples had the highest relative abundance (13%) of Acidobacteria, which was followed by the soil samples from N42 (10.5%), N47 (8.9%), N40 (4.7%), N44 (2.6%), and N45 (2.5%). Actinobacteria (Pearson correlation, *p* < 0.001) and Gemmatimonadetes (*p* < 0.001) were significantly enriched in high soil sodicity and salinity, while Acidobacteria (*p* < 0.05) and Proteobacteria (*p* < 0.001) were strongly suppressed by high pH and salt stress in the saline–sodic soils in our study.

At class level, Gamma-Proteobacteria, Alpha-Proteobacteria, Delta-Proteobacteria, Bacteroidia, Acidimicrobiia, Anaerolineae, Longimicrobia, Nitriliruptoria, Gemmatimonadetes, Chloroflexia, and Rhodothermia were the most abundant bacterial taxonomic groups in the soil samples. Gamma- and Delta-Proteobacteria were the two most abundant bacterial classes in N40 and N42 soil samples. The relative abundance of Delta-Proteobacteria was highest in N42 soils, while the relative abundance of Gamma-Proteobacteria was highest in N40 soils. The relative abundance of Longimicrobia was negligible (<0.1%) in N40 and N42 soils but ranged from 3.6% to 12.5% in the soils of the other four locations (N44: 12.5%; N47: 9.5%; N45: 5.8%; N46: 3.2%). Nitriliruptoria was also abundant in N44 (with a relative abundance of 9.8%) and N45 (8.4%) soils. The relative abundance of Chloroflexia was negligible in N40 and N42 soils but ranged from 1.6% to 3.6% in the other four locations. Pearson correlation analysis was performed to examine the relationships between different bacterial classes (Table S4). Positive interactions were observed among Gamma-Proteobacteria, Delta-Proteobacteria, Thermodesulfovibrionia, and Thermoanaerobaculia, with their relative abundances being positively correlated with each other (*p* < 0.05). These for bacterial classes were negatively influenced by increasing salinity. The bacterial classes, including Gamma-Proteobacteria, Delta-Proteobacteria, Bacteroidia, Thermoanaerobaculia, and Ignavibacteria, had a negative relationship with Nitriliruptoria and Rhodothermia. The correlation analysis also showed positive interactions among Acidimicrobiia, Nitriliruptoria, and Rhodothermia, which were significantly enriched in high salinity and sodicity.

The N44 and N45 soil samples had similar bacterial community composition, while N45 soil samples had higher bacterial community diversity and species richness than N44 soil samples. The bacterial community diversity and species richness in N44 and N45 soils were significantly lower than those in other samples. The N40 soils had similar bacterial community composition as N42 soils, as the soil samples from these two locations had the highest relative abundance of Proteobacteria.

### 3.4. Impacts of Soil Characteristics on Bacterial Community Composition

The correlation of bacterial taxonomic composition with soil physiochemical factors were tested to show the relationship between soil characteristics and bacterial community composition. The relative abundances of Gamma-Proteobacteria (Pearson correlation, *R* = −0.68, *p* = 0.02), Delta-Proteobacteria (*R* = −0.81, *p* < 0.001), Bacteroidia (*R* = −0.5, *p* = 0.036), and Thermomicrobia (*R* = −0.65, *p* = 0.0035) were negatively correlated with soil pH, while Nitriliruptoria (*R* = 0.94, *p* < 0.001) and Rhodothermia (*R* = 0.9, *p* < 0.001) were positively impacted by increasing soil pH (Figure S4). Increasing EC negatively impacted the relative abundances of Alpha-Proteobacteria (*R* = −0.56, *p* = 0.016), Gamma-Proteobacteria (*R* = −0.48, *p* = 0.042), and Bacteroidia (*R* = −0.69, *p* = 0.0015) but increased the relative abundances of Acidimicrobiia (*R* = 0.77, *p* < 0.001), Rhodothermia (*R* = 0.92, *p* < 0.001), and Nitriliruptoria (*R* = 0.9, *p* < 0.001; Figure S5). The content of Na^+^, K^+^, Ca^2+^, CO_3_^2−^, HCO_3_^−^, SO_4_^2−^, and Cl^−^ also significantly influenced the relative abundance of some key bacterial taxonomic groups. Specifically, the relative abundances of Acidimicrobiia, Rhodothermia, and Nitriliruptoria were positively correlated with the content of Na^+^, CO_3_^2-^, HCO_3_^−^, and Cl^-^ in the soil samples (Figure S6). The increasing content of Na^+^, CO_3_^2−^, and HCO_3_^−^ in soil negatively impacted the relative abundances of Gamma-Proteobacteria and Thermomicrobia. The relative abundance of Bacteroidia was positively correlated with the concentration of SO_4_^2−^ in soil (*R* = 0.59, *p* = 0.01), and Acidimicrobiia was positively influenced by increasing K^+^ concentration (*R* = 0.6, *p* = 0.0091).

### 3.5. Major Bacterial Genera Accounting for Community Composition Differences

**Figure 6:**
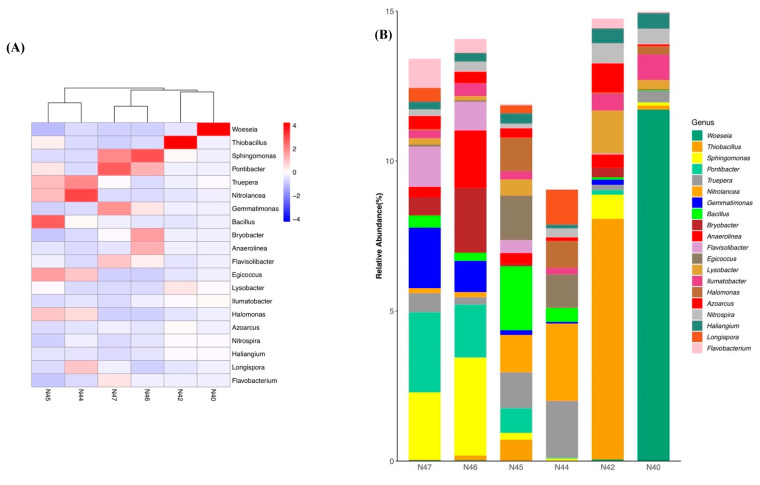
Bacterial community composition at genus level. (**A**) Heatmap clustering based on distribution patterns of 20 most abundant genus-level OTU groups. The dendrogram showing the cluster dissimilarity between samples was constructed based on maximum distance. (**B**) Bar plots showing the relative abundances of bacterial genera in saline soil bacterial communities.

Significant dissimilarities between the bacterial communities in different samples were shown in genus-level community composition. The dendrogram showing the cluster dissimilarity between samples revealed three clusters: N40 clustering with N42, N44 clustering with N45, and N46 clustering with N47 (Figure 6A). Both N44 and N45 soils had high relative abundance (>0.5%) of *Nitrolancea* (class: Thermomicrobia), *Bacillus* (class: Bacilli), *Truepera* (class: Deinococci), *Egicoccus* (class: Nitriliruptoria), and *Halomonas* (class: Gamma-Proteobacteria). Abundant *Sphingomonas* (class: Alpha-Proteobacteria), *Pontibacter* (class: Flavobacteriia), *Gemmatimonas* (class: Gemmatimonadetes), and *Flavisolibacter* (class: Chitinophagia) were detected in both N46 and N47 soils. The bacterial community in N40 soils was dominated by *Woeseia* (under the class of Gamma-Proteobacteria), while *Thiobacillus* (class: Beta-Proteobacteria) was the dominant bacterial genus in N42 soil bacterial community (Figure 6B). The relative abundance of *Woeseia* in N40 soils was up to 11.7%, and *Ilumatobacter* (1.3%; class: Acidimicrobiia), *Nitrospira* (1.1%; class: Nitrospira), and *Haliangium* (0.8%; class: Delta-Proteobacteria) were also abundant in N40 soils. *Thiobacillus* was the most abundant bacterial genus in N42 soils, with a relative abundance of 8.1%, followed by *Lysobacter* (1.4%; class: Gamma-Proteobacteria), *Azoarcus* (1%; class: Beta-Proteobacteria), *Sphingomonas* (0.8%), and *Nitrospira* (0.7%). The bacterial communities in N44, N45, N46, and N47 had relatively even abundance of the major bacterial genera within each soil sample, with the relative abundances of the most abundant bacterial genera below 4%. The most abundant bacterial genus in N44, N45, N46, and N47 was *Nitrolancea* (relative abundance in N44: 2.6%), *Bacillus* (relative abundance in N45: 2.1%), *Sphingomonas* (relative abundance in N46: 23.3%), and *Pontibacter* (relative abundance in N47: 2.7%), respectively.

**Figure 7:**
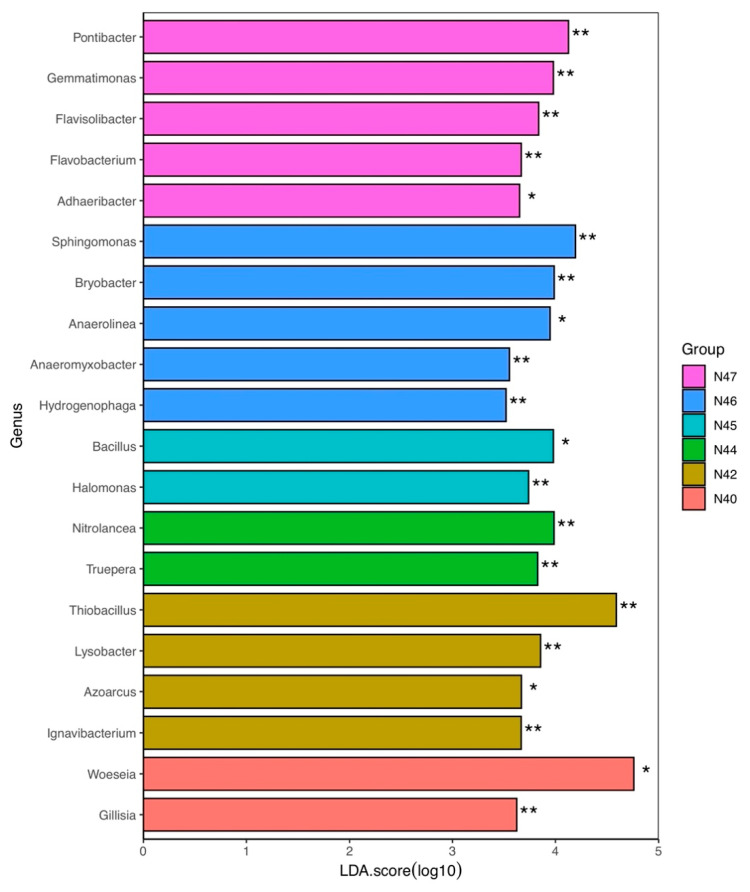
Difference in the bacterial community composition based on linear discriminant analysis effect size (LEfSe). Wilcoxon signed-rank test, Kruskal–Wallis H test, and linear discriminant analysis (LDA) were performed for determination of the OTU groups that most likely explain the differences between samples. The statistical significance of the effect is indicated by * *p* < 0.05 and ** *p* < 0.01.

The results of linear discriminant analysis effect size (LEfSe) showed the bacterial genera that most likely explain the differences in bacterial community composition between samples (Figure 7). The taxonomic groups that separated N40 from other samples were *Woeseia* (*p* < 0.05; Kruskal–Wallis test) and *Gillisia* (*p* < 0.01), while the differences of N42 were explained by *Thiobacillus* (*p* < 0.01), *Lysobacter* (*p* < 0.01), *Azoarcus* (*p* < 0.05), and *Ignavibacterium* (*p* < 0.01). *Nitrolancea* and *Truepera* accounted for the differences in N44 compared with other samples, and *Bacillus* and *Halomonas* were responsible for the differences in N45. The genera explaining the differences in N46 included *Sphingomonas*, *Bryobacter*, *Anaerolinea*, *Anaeromyxobacter*, and *Hydrogenophaga*, with the genera for N47 including *Pontibacter*, *Gemmatimonas*, *Flavisolibacter*, *Flavobacterium*, and *Adhaeribacter*.

## 4. Discussion

Broad-scale surveys are important for revealing the biogeographical patterns of soil bacterial communities and identifying the environmental factors influencing underground biodiversity. Previous studies have suggested significant differences in the structure of soil bacterial communities occur across the geographic distances, and climatic conditions and soil characteristics collectively determine soil bacterial biogeography [28,29]. However, soil bacterial biogeography is more explained by edaphic variables, such as soil pH, texture, organic matter content, and moisture, than climatic factors (e.g., latitude, site temperature, and precipitation) [30,31,32]. Geographic location showed notable impacts on the bacterial communities in our study, as the dendrogram showing the cluster dissimilarity between samples revealed three latitude-based clusters. Soil pH was the most significant factor in shaping bacterial community structure, suggesting that the soil characteristics were the major driving forces behind the observed biogeographical patterns. Past evolutionary events under natural selection and geographic distance isolation may also have contributed to the biogeographical patterns of the saline–sodic soil bacterial communities along the latitude gradients [33,34]. It is noteworthy that the geography can affect soil bacterial communities via impacts on soil genesis processes and other soil parameters [35,36]. The soil factors accounted for 58.58% of the total variations in bacterial community composition across all the soil samples in this study, and the remaining unexplained variations may be due to the latitude gradient and other unmeasured factors. For example, cover plants [37], soil redox conditions [38], management practices [39], organic matter mineralization [40], and biological interactions [41] have been reported to influence soil bacterial community patterns.

Excess salts and sodium levels are major characteristics of saline–sodic soils and can adversely affect soil structural stability, hydraulic conductivities, and infiltration rates [4,42]. The prevailing presence of salts and sodium leads to high pH and salt stress for living organisms in a soil system [43,44]. Soil pH ranged from 8.1 to 10.4, with EC ranging from 8.9 to 352 mS/cm across all samples along the latitude gradient in this study. The saline–sodic conditions imposed significant impacts on the soil bacterial community composition and diversity. Bacterial species richness and diversity significantly decreased with increasing pH, EC, and Na^+^ content. The results suggested that soil microbial diversity is negatively correlated with the salinity and sodicity in the environments, as increasing salt concentration may introduce ion toxicity and environmental pressures for inhibition of microbial enzyme activity and growth, thus imposing strong selection on microbial community composition [45,46,47]. The increased salinity in soil can elevate the extracellular osmolarity, which may damage cellular membrane and inactivate nucleic acids and proteins, thereby lysing the cell. Alternatively, the increased pH can also inhibit most members of the bacterial community, as the optimum pH range for bacterial activity and diversity is 6–7, out of which bacterial growth would be suppressed, leading to exclusion of specific bacterial groups under extreme conditions [48,49].

While high salinity may negatively influence a wide range of bacterial members, some bacterial taxonomic groups may have developed strategies to adapt to the harsh environments. The bacterial groups that are resistant to high salinity and pH could be enriched and prevalent in saline soils. By examining the bacterial community composition, we showed that the dominant phyla in the saline–sodic soils included Proteobacteria (dominant classes: Gamma-, Alpha-, and Delta-Proteobacteria), Actinobacteria (Acidimicrobium and Nitriliruptoria), Gemmatimonadetes (Longimicrobia), Chloroflexi (Anaerolinea), Bacteroidetes, Acidobacteria, and Firmicutes. Proteobacteria was specifically enriched in N40 and N42 soils, with the relative abundances up to 50% in our study, while Gemmatimonadetes and Actinobacteria had higher relative abundance in N44, N45, N46, and N47 than other types of soils. Gemmatimonadetes, with the ability to adapt to low soil moisture, are widely distributed in natural environments and especially abundant in a variety of arid soils, but Gemmatimonadetes generally have low relative abundance, around 2%, in soil [50,51,52]. However, the relative abundance of Gemmatimonadetes was as high as 16% in the saline–sodic soils in this study, suggesting high salinity–sodicity tolerance. Other studies showed that the relative abundance of Gemmatimonadetes was negatively correlated with organic nutrients but positively correlated with pH and Na^+^ concentration in soil [14,19,52]. Proteobacteria accounted for the largest proportion of the bacterial community across all the saline–sodic soil samples, which indicates high metabolic capability and bacterial growth under high salinity levels. In particular, Alpha- and Gamma-Proteobacteria were enriched in high-salinity soils. However, the relative abundance of Proteobacteria was found to be inversely correlated to soil pH over the pH value range of 8.2 to 10.1 [52], which may explain the results that the relative abundance of Proteobacteria was highest in N40 and N42 soils and decreased in other soil samples in response to the increase in pH. Actinobacteria, as the second most abundant bacterial phylum, were specifically enriched in soils of high salinity and sodicity, i.e., N44 and N45 soils in this study. The relative abundance of Actinobacteria was positively correlated with soil pH and Na^+^ concentration, representing high adaptation capacity to high salinity and sodicity. Actinobacteria are widely distributed in soil and especially predominant in dry desert soils, and they have critical roles in terrestrial ecosystem functioning, for example, contributing to global carbon cycling and plant productivity via soil organic matter decomposition and synthetization of bioactive compounds [46,53]. Acidimicrobiia and Nitriliruptoria were the most abundant classes under Actinobacteria, both of which showed high resilience to the extreme conditions of high pH and salt stress, with their relative abundances being positively associated with soil pH and salinity levels. Our study suggested that Actinobacteria can adapt well to high salinity–sodicity and were highly abundant in saline–sodic soils and are important in sustaining the saline–sodic soil ecosystem function. The highly abundant bacterial taxonomic groups may be potential biological resources for reclamation of the degraded soil system.

The interactions between different bacterial taxonomic groups were also an important role in structuring the bacterial communities in the saline soils. For example, Gamma-Proteobacteria, Delta-Proteobacteria, Thermodesulfovibrionia, and Thermoanaerobaculia had positive interactions with each other but may have been negatively impacted by the enriched bacterial classes (e.g., Acidimicrobiia, Nitriliruptoria, and Rhodothermia) in high salinity and sodicity. Most bacterial classes were highly connected with other bacterial groups, suggesting that bacterial interactions within the adverse conditions of saline soils may significantly influence the survival and function of soil bacterial community members.

Despite large numbers of bacterial taxa shared among different sites, notable variations in bacterial community composition were revealed within the six saline–sodic soils across the broad latitude gradient, suggesting strong local selections over bacterial assembly. Previous studies have shown intense habitat specialization for bacterial community structure in the bacterial community assembly processes across different geographic locations [47,54,55]. Xun et al. [56] showed that bacterial community diversity was also correlated with the assembly processes of bacterial communities, and deterministic processes tended to dominate low-diversity communities. The bacterial community diversity and species richness in the saline soils of this study were lower compared with other agricultural soils; thus, the saline soil bacterial community may be more subject to the environmental factors at local habitat. Our results suggest that niche-based environmental characteristics and biotic interactions that select subsets of bacterial taxonomic groups from a regional species pool were a major driving force of bacterial community assembly. The indicator taxa that were predominant in each site varied across all soils, and the members of Gamma- and Alpha-Proteobacteria were highly represented in N40 (i.e., *Woeseia* and *Gillisia*) and N42 (i.e., *Thiobacillus*, *Lysobacter*, *Azoarcus*, and *Ignavibacterium*) soils. *Nitrolancea* and *Truepera* (dominant in N44), along with *Bacillus* and *Halomonas* (N45), were greatly enriched in soils of high salinity and sodicity. The indicator taxa that separated the N46 soil bacterial community from others included *Sphingomonas*, *Bryobacter*, *Anaerolinea*, *Anaeromyxobacter*, and *Hydrogenophaga*, with *Pontibacter*, *Gemmatimonas*, *Flavisolibacter*, *Flavobacterium*, and *Adhaeribacter* accounting for the differences in N47 soil bacterial community. All these bacterial taxonomic groups were widely distributed across saline soils [19,30,31,55]; however, more efforts are needed to reveal their distribution patterns in a broad range of salinity and sodicity and investigate their ecological roles in the salt-affected soil systems. Soil salinization significantly alters soil bacterial community diversity and taxonomic composition. Thus, identifying indicator taxa of saline soil bacterial communities may be useful for reflecting changes in soil quality. However, only 18 samples along the latitude gradient were analyzed, and the conclusions about the geographic patterns and assembly of bacterial communities in this study are not widely applicable and may only be applicable to soils with similar properties.

Techniques for mitigation and restoration of saline soils, such as salt leaching, flushing, organic amendment, microbial remediation, and agricultural practices, have been proposed and adopted for practical application. Our study suggests that microbial community features and indicator taxa may serve as important evaluation indicators for soil health conditions. The results indicate that the bacterial taxa that can best adapt to high salinity, such as *Bacillus*, *Egicoccus*, *Truepera*, and *Nitrolancea*, may have potential to be used in microbial remediation of saline soils in combination with organic amendment and other practices.

## 5. Conclusions

Our study revealed evident biogeographical patterns of bacterial communities in the saline oil ecosystems along a broad latitude gradient in the Northeast China Plain. Here, we presented a field study of the saline soil ecosystems along a broad latitude gradient in the Northeast China Plain. Our results revealed evident biogeographical patterns of soil bacterial communities and showed the predominant indicator taxa at each site. Soil salinity and sodicity were the primary soil factors behind the habitat selection over bacterial communities, explaining 58.58% of the total variations in bacterial community composition across all the soil samples in this study. Increased soil pH significantly decreased bacterial species richness and diversity in the soils of this study. The bacterial phyla Actinobacteria and Gemmatimonadetes were significantly enriched in high soil sodicity and salinity, while Acidobacteria and Proteobacteria were suppressed by high pH and salt stress in the saline–sodic soils. The predominant indicator taxa, i.e., *Bacillus*, *Egicoccus*, *Truepera*, *Nitrolancea*, *Nitrolancea*, and *Halomonas*, in the saline soils may be potentially applied in combination with other measures, such as addition of organic matter and use of protective crops for remediation of saline soils. These results collectively suggested that niche-based environmental characteristics and biotic interactions are the most major driving force in shaping soil bacterial community structure, even in a broad biographic gradient. The profiling of the saline soil bacterial communities along the latitude gradient will also provide a basis for restoration of salt-affected soil ecosystems.

## Data Availability

The raw bacterial 16S rRNA gene sequences generated in this study were uploaded to Sequence Read Archive (SRA) at NCBI and can be accessed via accession number of PRJNA752985.

## Supplementary Materials

The following supporting information can be downloaded at: https://www.mdpi.com/article/10.3390/microorganisms10091787/s1, Table S1: The geographic locations of the collected soil samples in Northeast China along a south-north latitudinal gradient (40°53′ N to 47°26′ N); Table S2: The physiochemical properties of soil samples collected from saline soils in Northeast China along a latitude gradient; Table S3: Pearson correlation of bacterial community diversity (Shannon index) and species richness (Chao1 index) with soil factors; Table S4: Relationships between different bacterial groups. Pearson correlation between relative abundances of different members was performed. Red color represents negative interactions between the two bacterial taxa, and blue color denotes positive interactions. Significance is indicated by * *p* < 0.05, ** *p* < 0.01, *** *p* < 0.001, no symbol indicates no significant correlation (*p* > 0.05); Figure S1: Heatmap of the soil physiochemical properties. The asterisks indicate the significant correlation between different soil characteristics as revealed by Spearman’s correlation analysis (* *p* < 0.05; ** *p* < 0.01); Figure S2: Rarefaction curve with maximum read depth. Sufficient sequencing depth was achieved for representative coverage of the bacterial community diversity in each sample; Figure S3: The bacterial community composition at phylum and class level; Figure S4: Pearson correlation of bacterial taxonomic composition with soil pH; Figure S5: Pearson correlation of bacterial taxonomic composition with soil EC; Figure S6: Pearson correlation of bacterial taxonomic composition with soil physiochemical factors including the content of Na^+^, K^+^, Ca^2+^, CO_3_^2−^, HCO_3_^−^, SO_4_^2−^, and Cl^−^.
