# Supplementary material for: Biogeographical Patterns and Assembly of Bacterial Communities in Saline Soils of Northeast China
Source: Microorganisms. 2022 Sep 5;10(9):1787. doi: 10.3390/microorganisms10091787 (PMC9505542; doi:10.3390/microorganisms10091787)
Supplement: Supplementary file 1 [file microorganisms-10-01787-s001.zip › microorganisms-1886632-supplementary.pdf]

Supplementary Information

Biogeographical patterns and assembly of bacterial communities in saline soils of Northeast China

Xiaolong Liang[1#], Xiaoyu Wang[2#], Ning Zhang[3*], Bingxue Li[2*]

[1] Key Laboratory of Pollution Ecology and Environmental Engineering, Institute of Applied Ecology, Chinese Academy of Sciences, Shenyang 110016, China

[2] College of Land and Environment, Shenyang Agricultural University, Shenyang 110866, China

[3] College of Bioscience and Biotechnology, Shenyang Agricultural University, Shenyang 110866, China

# The authors contributed equally to this study.

* Correspondence: zhangning@syau.edu.cn; libingxue@syau.edu.cn.

Table S1. The geographic locations of the collected soil samples in Northeast China along a south-north latitudinal gradient (40°53′ N to 47°26′ N).

Sample	Altitude	Latitude (N)	Longitude (E)	pH
N47A	149.55m	47°25′49″	123°35′24″	9.21
N47B	150.48m	47°26′24″	123°35′55″	9.22
N47C	148.93m	47°26′45″	123°36′34″	8.94
N46A	133.15m	46°05′35″	123°15′44″	8.5
N46B	132.03m	46°05′11″	123°15′22″	8.8
N46C	133.97m	46°04′49″	123°15′42″	8.62
N45A	127.27m	45°43′58″	124°45′57″	10.45
N45B	128.01m	45°44′15″	124°46′18″	10.43
N45C	127.36m	45°44′20″	124°46′29″	10.44
N44A	140.16m	44°30′24″	123°08′09″	10.24
N44B	141.06m	44°30′33″	123°08′19″	10.35
N44C	139.38m	44°31′39″	123°08′58″	10.37
N42A	251.28m	42°47′34″	122°26′32″	8.4
N42B	250.64m	42°49′53″	122°24′38″	8.35
N42C	249.28m	42°49′50″	122°24′55″	8.39
N40A	20.09m	40°53′49″	121°50′02″	8.13
N40B	20.33m	40°53′30″	121°49′51″	8.19
N40C	20.49m	40°53′21″	121°49′39″	8.16

Note: Soil samples were collected from six sites across saline agricultural soils of three provinces in Northeast China. The letter N in the sample name represents North latitude and the number represents the degree of latitude. N40, N42, N44, N45, N46, and N47 represent the latitude degree of each sampling site. The letters A, B, and C suggest that the samples are three replicates sampled from each site.

Table S2. The physiochemical properties of soil samples collected from saline soils in Northeast China along a latitude gradient.

Sample	EC (mS/cm)	Ca^{2+} (g/kg)	Mg^{2+} (g/kg)	K^{+} (g/kg)	Na^{+} (g/kg)	CO_3^{2-} (g/kg)	HCO_3^{-} (g/kg)	Cl^{-} (g/kg)	SO_4^{2-} (g/kg)
N47A	78.9	0.43	0.06	0.04	3.5	0.94	1.44	1.44	7.63
N47B	109.2	0.46	0.07	0.04	3.86	1.95	0.75	1.25	6.67
N47C	64.1	0.4	0.1	0.03	2.04	1.64	0.98	0.87	7.58
N46A	18.59	0.31	0.11	0.02	0.61	0	1.17	0.22	3.81
N46B	16.5	0.24	0.09	0.02	0.41	0	1.04	0.16	2.94
N46C	15.5	0.31	0.04	0.02	0.54	0	0.74	0.13	3.28
N45A	366	0.27	0.07	0.03	4.98	0.67	9.01	5.38	2.26
N45B	337	0.31	0.07	0.03	4.7	2.41	8.14	5.94	2.39
N45C	335	0.33	0.09	0.04	5.72	1.3	5.42	5.58	2.29
N44A	295	0.13	0.03	0.03	4.43	1.59	5.78	4.73	3.35
N44B	372	0.14	0.02	0.03	4.95	0.75	6.89	5.07	2.81
N44C	390	0.19	0.04	0.04	4.47	1.37	8.13	2.89	2.71
N42A	6.9	0.2	0.01	0.01	0.1	0	0.62	0.32	4.08
N42B	11.44	0.25	0.04	0.03	0.19	0	0.85	0.17	3.88
N42C	8.24	0.21	0.03	0.02	0.17	0	1.12	0.24	2.32
N40A	139.4	0.09	0.05	0.09	2.13	0	1.1	3.3	2.64
N40B	245	0.3	0.23	0.36	3.81	0.17	1.05	5.04	2.57
N40C	137.4	0.32	0.08	0.13	2.29	0	1.01	3.56	2.68

Table S3. Pearson correlation of bacterial community diversity (Shannon index) and species richness (Chao1 index) with soil factors.

	Chao1		Shannon	
	r	*P*	*r*	*P*
pH	-0.919586	0.000006816	-0.9016787	3.213E-07
EC	-0.8356093	0.00001579	-0.8917889	6.698E-07
Ca^{2+}	0.2362864	0.3452	0.27	0.2761
Mg^{2+}	0.23	0.3591	0.219	0.3829
K^{+}	0.19	0.46	0.11	0.66
Na^{+}	-0.776	0.000152	-0.84	0.00001281
CO_3^{2-}	-0.67	0.002448	-0.63	0.005283
HCO_3^{-}	-0.86	0.00000539	-0.88	0.000001598
Cl^{-}	-0.65	0.003454	-0.75	0.0003483
SO_4^{2-}	0.23	0.3537	0.26	0.3029

Table S4. Relationships between different bacterial groups. Pearson correlation between relative abundances of different members was performed. Red color represents negative interactions between the two bacterial taxa, and blue color denotes positive interactions. Significance is indicated by $*p < 0.05$, $**p < 0.01$, $***p < 0.001$, no symbol indicates no significant correlation ($p > 0.05$).

	Gammaproteobacteria	Deltaproteobacteria	Acidimicrobiia	Alphaproteobacteria	Bacteroidia	Anaerolineae	Thermoanaerobaculia	Rhodothermia	Nitriliruptoria	Thermodesulfovibrionia	Gemmatimonadetes	Ignavibacteria
Gammaproteobacteria		0.98***	-0.14	-0.11	0.068	0.29	0.74***	-0.63**	-0.67**	0.67**	-0.52*	0.66**
Deltaproteobacteria	0.98***		-0.1	-0.31	-0.07	0.36	0.77***	-0.51*	-0.54*	0.79***	-0.6**	0.7**
Acidimicrobiia	-0.14	-0.1		-0.44	-0.62**	-0.5*	-0.19	0.66**	0.58*	-0.43	-0.47**	-0.57*
Alphaproteobacteria	-0.11	-0.31	-0.44		0.61***	0.08	0.02	-0.47*	-0.46	-0.35	0.55*	-0.05
Bacteroidia	0.068	-0.07	-0.62**	0.61***		0.12	-0.04	-0.73***	-0.69**	-0.11	0.58*	0.03
Anaerolineae	0.29	0.36	-0.5*	0.08	0.12		0.3	-0.38	-0.34	0.61**	-0.23	0.66**
Thermoanaerobaculia	0.74***	0.77***	-0.19	0.02	-0.04	0.3		-0.54*	-0.57*	0.67**	-0.27	0.61**
Rhodothermia	-0.63**	-0.51*	0.66**	-0.47*	-0.73***	-0.38	-0.54*		0.98***	-0.43	-0.24	-0.56*
Nitriliruptoria	-0.67**	-0.54*	0.58*	-0.46	-0.69**	-0.34	-0.57*	0.98***		-0.41	-0.21	-0.54*
Thermodesulfovibrionia	0.67**	0.79***	-0.43	-0.35	-0.11	0.61**	0.67**	-0.43	-0.41		-0.33	0.91***
Gemmatimonadetes	-0.52*	-0.6**	-0.47**	0.55*	0.58*	-0.23	-0.27	-0.24	-0.21	-0.33		-0.19
Ignavibacteria	0.66**	0.7**	-0.57*	-0.05	0.03	0.66**	0.61**	-0.56*	-0.54*	0.91***	-0.19	

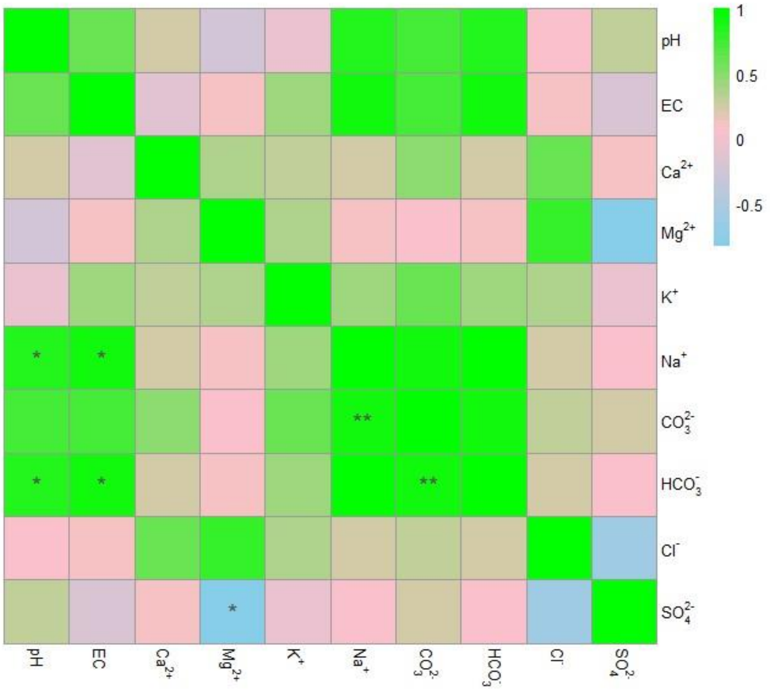

Figure S1. Heatmap of the soil physiochemical properties. The asterisks indicate the significant correlation between different soil characteristics as revealed by Spearman's correlation analysis (* $P<0.05$; ** $P<0.01$).

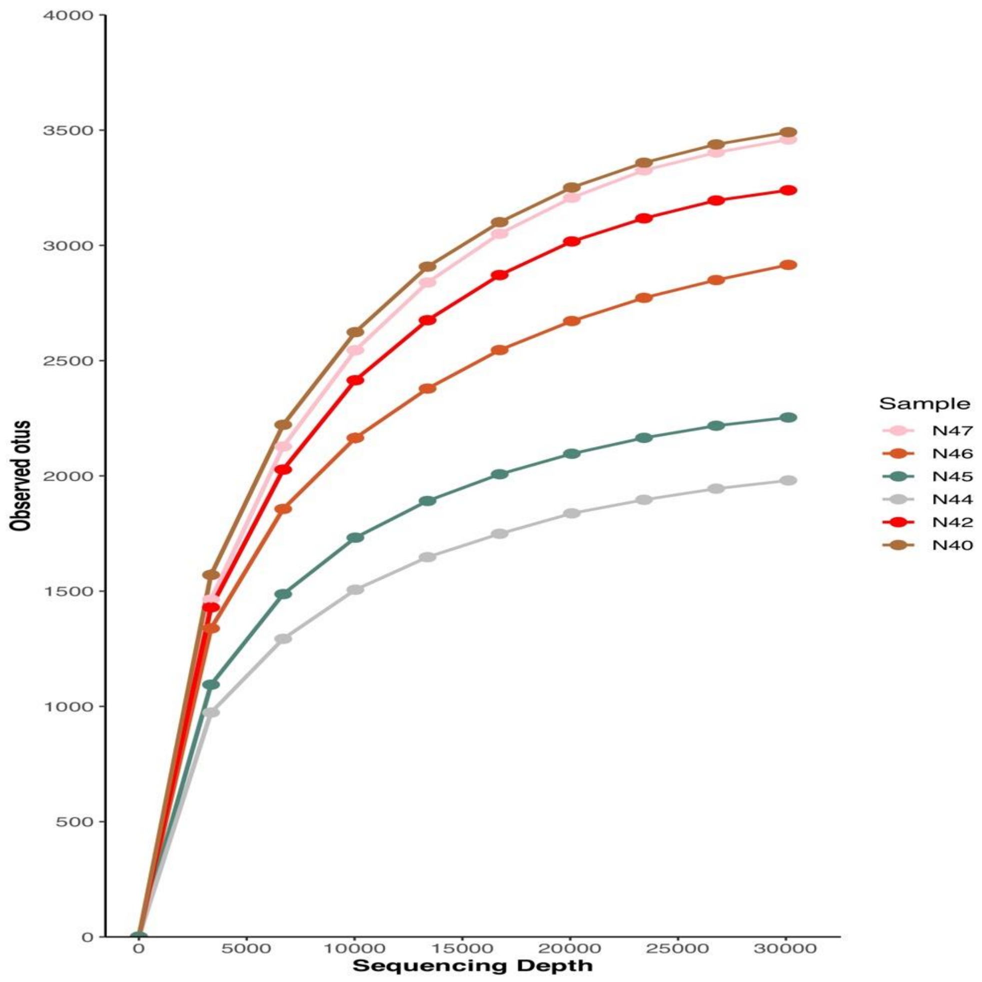

Figure S2. Rarefaction curve with maximum read depth. Sufficient sequencing depth was achieved for representative coverage of the bacterial community diversity in each sample.

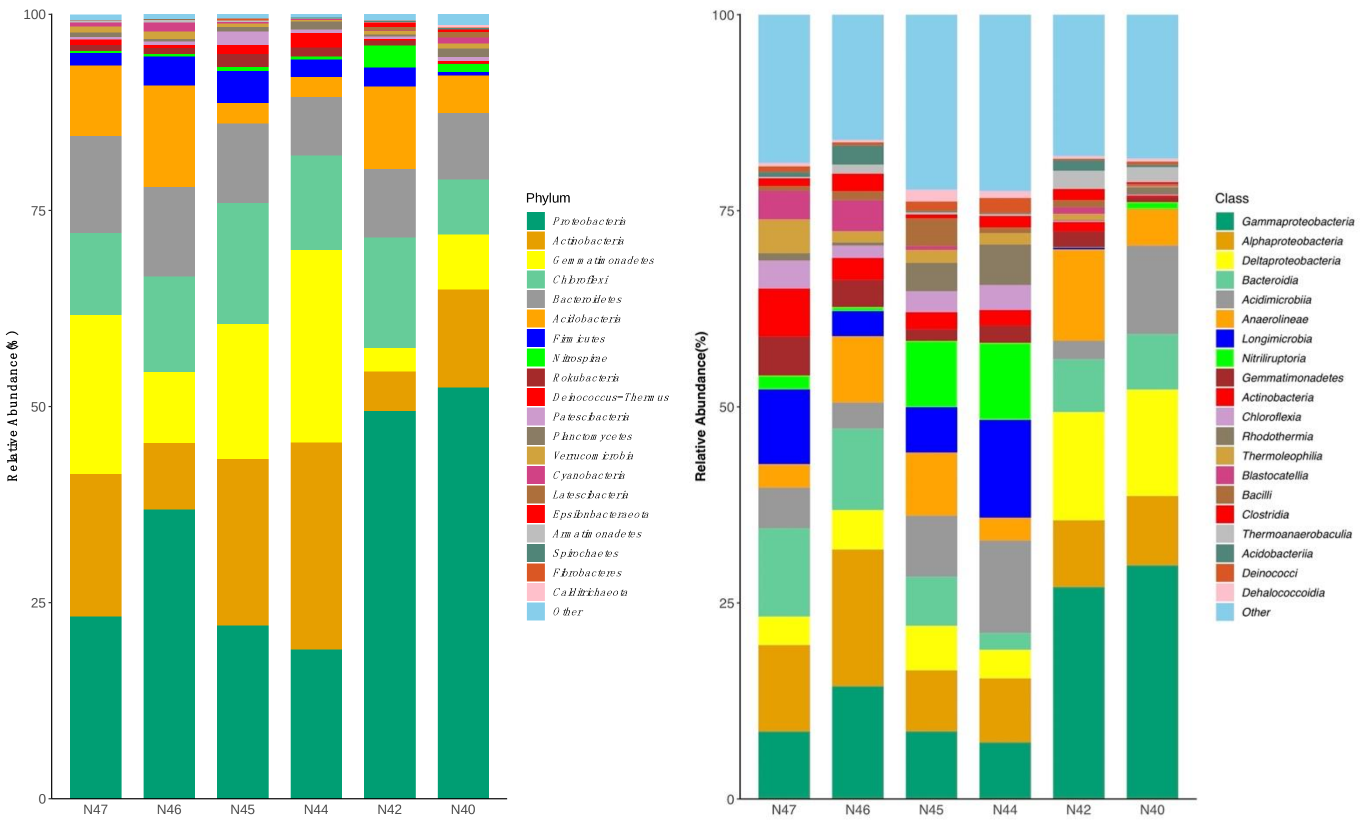

Figure S3. The bacterial community composition at phylum and class level.

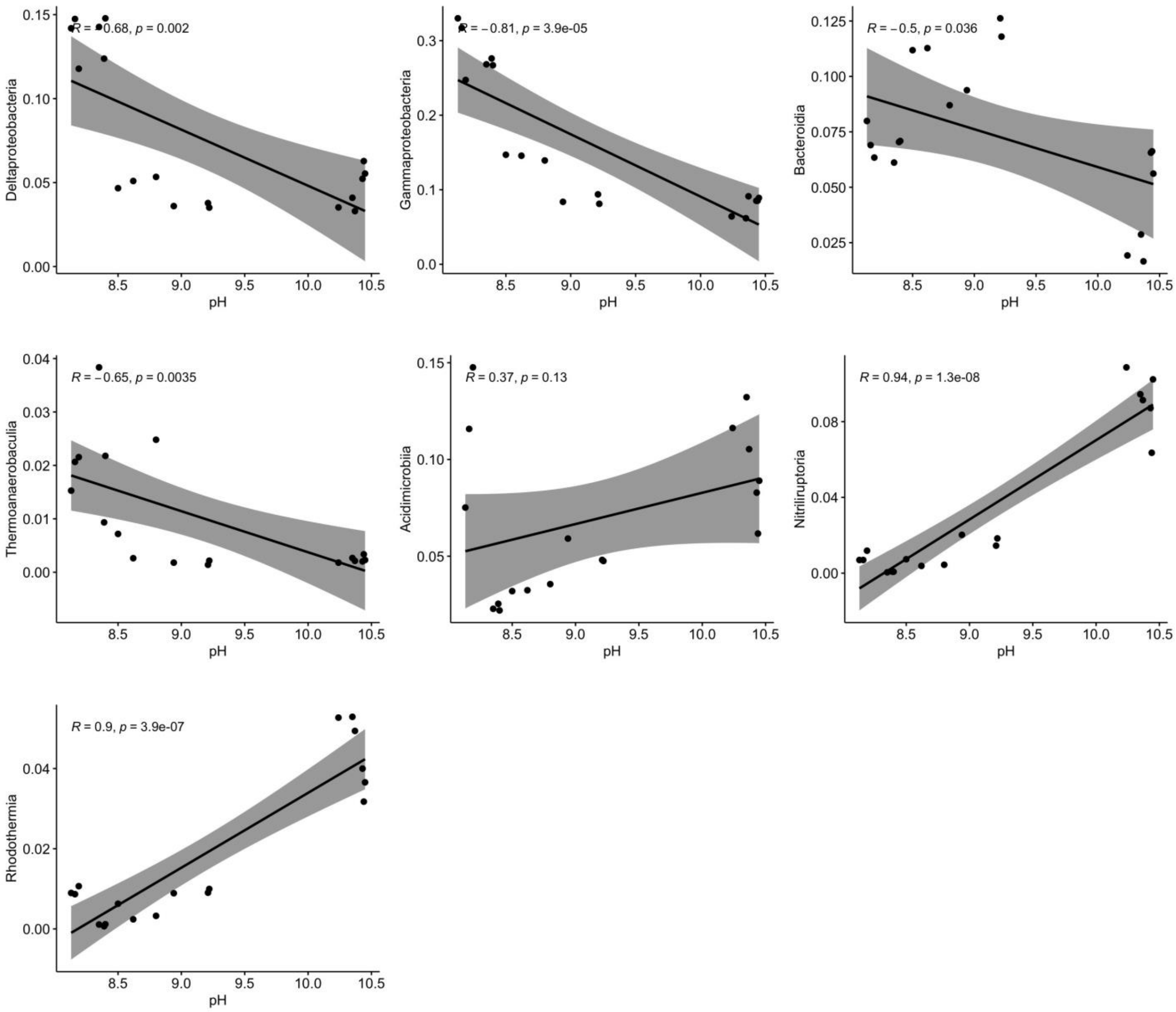

Figure S4. Pearson correlation of bacterial taxonomic composition with soil pH.

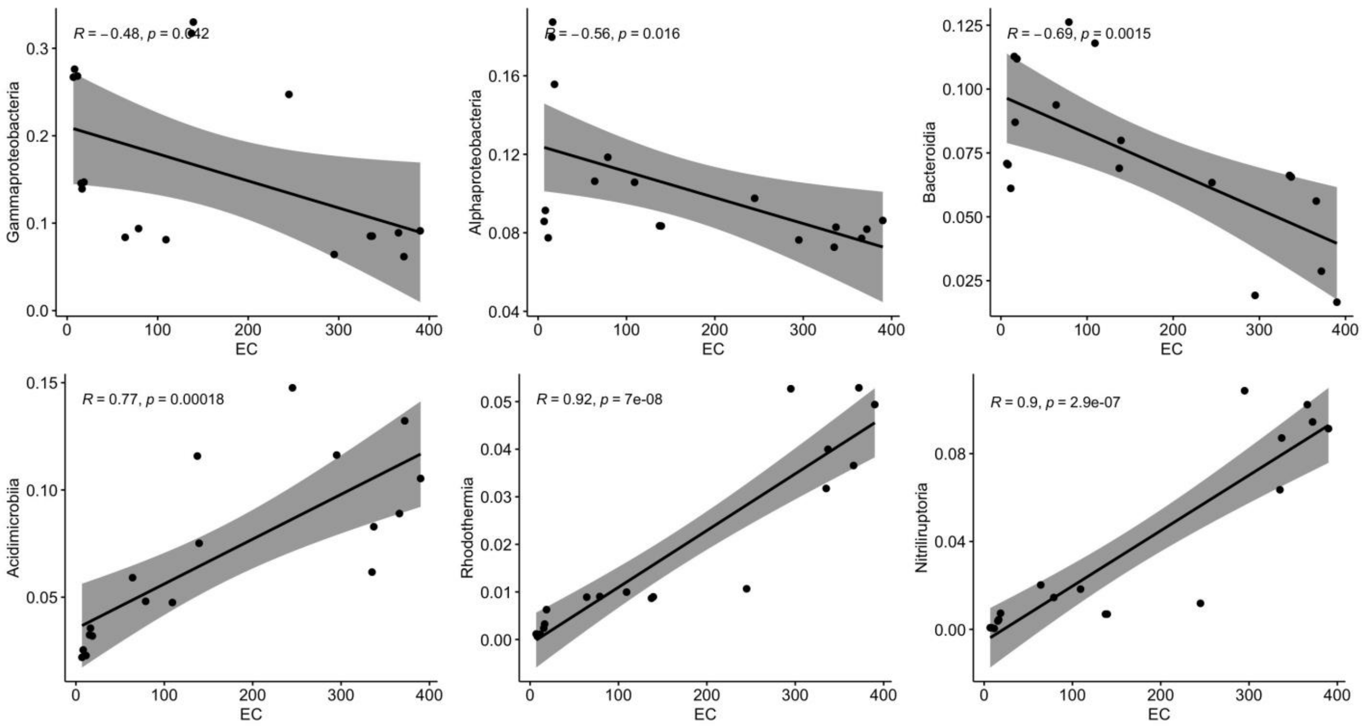

Figure S5. Pearson correlation of bacterial taxonomic composition with soil EC.

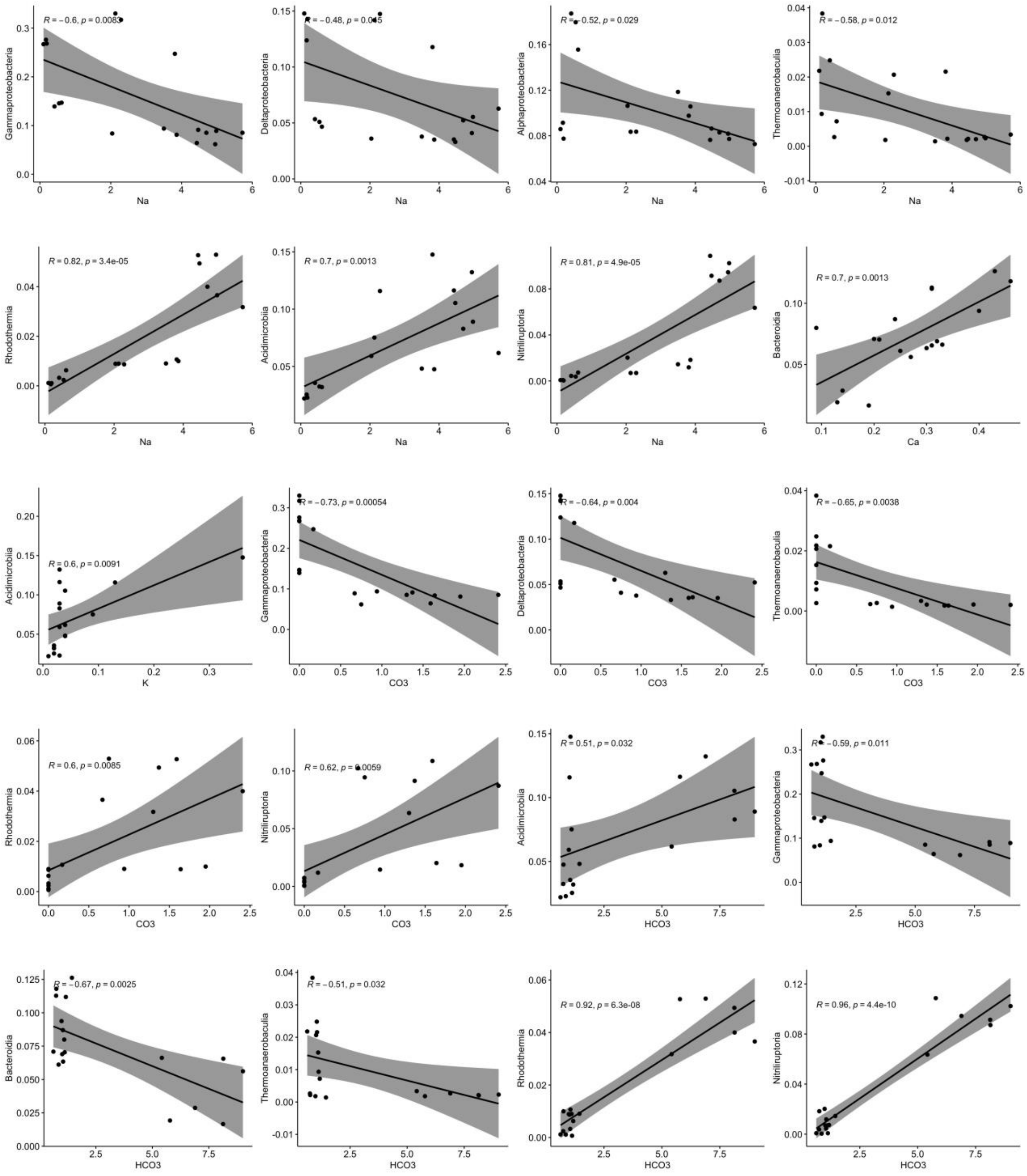

R = −0.6, p = 0.0083
Gammaproteobacteria
Na
Deltaproteobacteria
R = −0.52, p = 0.029
Alphaproteobacteria
R = −0.58, p = 0.012
Thermoanaerobaculia
R = 0.82, p = 3.4e-05
Rhodothermia
R = 0.7, p = 0.0013
Acidimicrobiia
R = 0.81, p = 4.9e-05
Nitriliruptoria
R = 0.7, p = 0.0013
Bacteroidia
Ca
R = 0.6, p = 0.0091
K
R = −0.73, p = 0.00054
CO3
R = −0.64, p = 0.004
R = −0.65, p = 0.0038
R = 0.6, p = 0.0085
R = 0.62, p = 0.0059
R = 0.51, p = 0.032
HCO3
R = −0.59, p = 0.011
R = −0.67, p = 0.0025
R = −0.51, p = 0.032
R = 0.92, p = 6.3e-08
R = 0.96, p = 4.4e-10

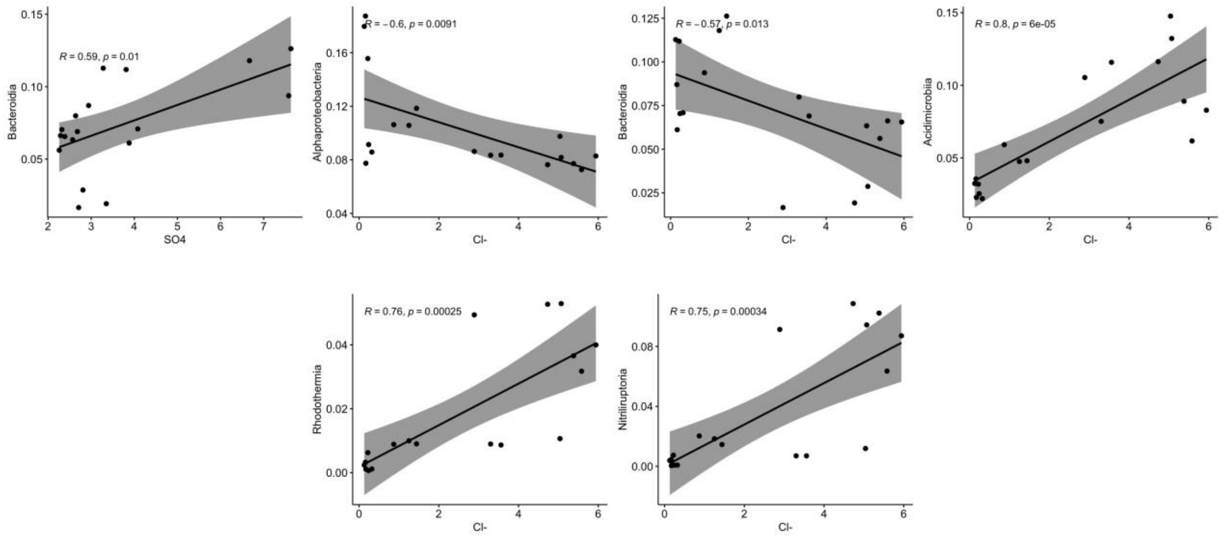

Figure S6. Pearson correlation of bacterial taxonomic composition with soil physiochemical factors including the content of Na^{+}, K^{+}, Ca^{2+}, CO_3^{2-}, HCO_3^{-}, SO_4^{2-}, and Cl^{-}.
